# Perioperative Immunosuppression and Risk of Cancer Progression: The Impact of Opioids on Pain Management

**DOI:** 10.1155/2018/9293704

**Published:** 2018-09-19

**Authors:** Renata Zajączkowska, Wojciech Leppert, Joanna Mika, Magdalena Kocot-Kępska, Jarosław Woroń, Anna Wrzosek, Jerzy Wordliczek

**Affiliations:** ^1^Department of Interdisciplinary Intensive Care, Jagiellonian University Medical College, Kraków, Poland; ^2^Department of Palliative Medicine, Poznan University of Medical Sciences, Poznan, Poland; ^3^Department of Pain Pharmacology, Institute of Pharmacology, Polish Academy of Sciences, Kraków, Poland; ^4^Department of Pain Research and Treatment, Jagiellonian University Medical College, Kraków, Poland

## Abstract

Opioids comprise an important group of drugs used in cancer pain pharmacotherapy. In recent years, more and more studies have emerged indicating the potentially immunosuppressive effects of opioid analgesics and their serious consequences, including the risk of cancer progression. The identification of these risks has prompted a search for other effective, and most importantly, safer methods of perioperative analgesic management. Regional analgesia techniques, which allow for a significant reduction in opioid dosing and thus diminish the risk of immunosuppression associated with these drugs, seem to offer substantial hope in this respect. A number of studies available in the literature assess the effects of regional analgesia techniques on cancer progression; however, it is often difficult to interpret their results owing to several perioperative factors (such as surgical trauma, inadequate pain and stress relief, and hypothermia) which are also attributed immunosuppressive effects and tend to be implicated in increased risk of cancer progression. Further research is needed to verify the available data on both the potential adverse effects of opioids and the possible protective effects of regional analgesia techniques on cancer patients.

## 1. Introduction

Cancer pain poses a serious clinical and epidemiological problem. It may be the first symptom of the disease, occurs during its diagnosis and treatment, and accompanies patients at the advanced stages of the disease. It is estimated that, regardless of the stage, at least one-half of cancer patients experience pain, but their proportion is significantly higher as the disease progresses. The World Health Organisation (WHO) reports that over 5.5 million patients worldwide do not receive or receive inadequate treatment for cancer-related pain. The above data, due to the lack of worldwide registers, are based on epidemiological estimates [1]. A meta-analysis published in 2016, which included 117 reports of pain in cancer patients, showed that the goal of effective pain management still remains far from being achieved: pain was experienced by 39.3% of patients after radical cancer treatment, by 55% of patients during cancer treatment, and by 66.4% at the advanced stage of cancer treatment. 38% of patients experienced moderate to severe pain (NRS > 5) [2].

Apart from the unimaginable suffering and the humanitarian aspect of the problem, the consequences of insufficiently treated pain, both acute and chronic, can also be disastrous from a purely medical point of view. This is particularly important in cancer patients as more and more data indicate a correlation between severe pain and increased risk of cancer progression and a shorter time to the appearance of metastatic lesions [3]. Research shows that ineffective pain and stress treatment adversely affects the body's defence systems, including cellular immunity and the functioning of the natural killer (NK) cells. Andersen et al. demonstrated that the stress associated with the diagnosis and surgical treatment of breast cancer in women impaired their immune cell response, including NK cell toxicity and T-cell responses. Stress levels significantly predicted lower NK cell lysis, diminished response of NK cells to recombinant interferon gamma, and decreased proliferative response of peripheral blood lymphocytes to plant lectins and to a monoclonal antibody directed against the T-cell receptor [4].

Experimental and clinical studies demonstrate the importance of effective pain management also in terms of cancer progression. Page et al. found a longer time interval to lung metastasis in a group of experimental animals (rats in the experimental model of breast adenocarcinoma) with effective analgesia (intrathecal or systemic opioids) compared with a group of animals not subject to analgesic treatment [5]. Lillemoe et al. in their randomised prospective study (RCT), which included a group of patients with advanced pancreatic cancer, concluded that alcohol-induced visceral neurolysis (neurolytic splanchnicectomy) performed in one of the two groups of patients provided not only pain relief but also longer survival time, compared with the group of patients given saline instead of alcohol. There was no improvement in overall survival (OS) compared with the control group of patients who underwent the same procedure but who did not experience pain prior to its onset [6].

Opioids are being used by the anaesthesiologists not only to treat acute pain in the perioperative period but also to control chronic cancer pain. Studies conducted over the recent years have given results indicating the potentially immunosuppressive effects of opioids and their serious consequences, including the risk of cancer progression. This is a very important and complex problem both in perioperative period and in chronic pain treatment course. Morphine has immunosuppressant properties which can promote cancer, but on the other hand suppressing of pain alleviates the surgical stress and thus might be protective against tumour metastases.

## 2. Opioids and Their Receptors

Pharmacotherapy constitutes the basic pain treatment method in cancer patients. For moderate to severe pain in cancer patients, the mainstay of the therapy is opioid analgesics (weak or strong ones, depending on pain severity). These drugs exert their clinical effects by influencing individual opioid receptors, which are currently classified into three groups: MOP (*µ*, mu), DOP (*σ*, delta), and KOP (*κ*, kappa). Opioids can also affect other receptors, especially NOPs (nonclassical nociceptin/orphanin FQ receptor, N/OFQ). In everyday practice, depending on the type of pain (receptor: somatic and visceral or neuropathic), the clinical condition of the patient, the coexisting diseases, and capacities of organs crucial for drug metabolism and excretion (liver and kidneys), different opioids are used, including morphine, oxycodone, hydromorphone, fentanyl, buprenorphine, tapentadol, methadone, tramadol, codeine; the last two are being classified as the so-called “weak” opioids.

Owing to the steadily increasing use of opioids, their potential side effects are being studied in more depth. In recent years, a number of studies have suggested immunosuppressive effects of opioid analgesics and their potential serious consequences, including an increased risk of cancer progression in patients treated with these drugs [3].

Classically, three opioid receptor types (*µ*, *σ*, and *κ*) have been identified on the central and peripheral nervous system neurons. Moreover, based on the analysis of mRNA, receptor protein, or opioid binding capacity of cells or their opioid response, it has been confirmed that opioid receptors are also present on other cells, including cancer cells [7]. Interestingly, it has been shown that, by influencing the proliferation and apoptosis processes, opioids can regulate the growth and activity of a number of cells, including the cancer cells [8]. This may result in an increased risk of cancer progression. However, the test results are ambiguous as they depend on the type of cell tested, the dosage, the time of administration, and the type of opioid used. The question remains whether the direct effect of opioids on cancer cells observed in experimental studies translates into comparable effects in vivo and how opioids ultimately affect the immune system status of cancer patients.

## 3. Potential Mechanisms of Opioid-Induced Immunosuppression

**Figure 1:**
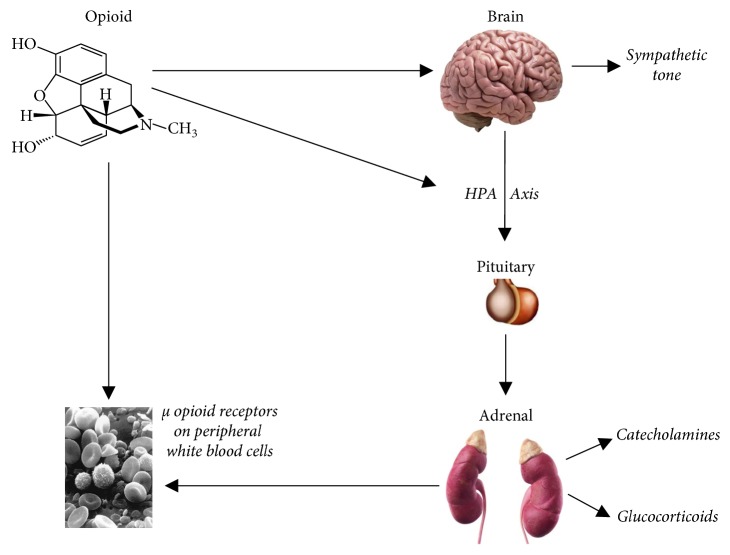
Potential mechanisms of immunosuppressive effects of opioids [13].

The immune system plays a key role in cancer defence. Its important components include NK (natural killer cells), T cells, mast cells, macrophages and mediators, including cytokines (interleukins and chemokines). It turns out that acute and chronic administration of exogenous opioids affects both the cellular and humoral components of the immune response. Exogenous opioids affect several of the components of the immune system, including lymphocyte proliferation, their phagocytic activity, NK cell activity, cytokine expression, and antibody production [9]. Several mechanisms responsible for immunosuppression associated with the use of opioids in cancer patients have been identified. They can be divided into central and peripheral ones and are schematically presented in Figure 1.

The importance of central mechanisms is demonstrated by the fact that opioids that easily penetrate the blood-brain barrier have a stronger immunosuppressive effect compared with those that do not pass through it—the latter demonstrates this effect only after central administration [10]. Moreover, it turned out that MOP knockout mice did not show any immune-modulating effect after central administration of morphine in experimental studies. This confirms the view that central immune modulatory effects of opioids are mediated by the MOP receptor [11]. A central site for opiate action in the induction of immunosuppression appears to be the periaqueductal gray matter (PAG) which also subserves a variety of diverse autonomic functions. Microinjections of morphine into the PAG result in a rapid suppression of natural killer (NK) cell activity [12].

The peripheral mechanisms most likely involve the activation of MOP opioid receptors located on immunocompetent cells [13]. Opioid receptors have been identified on the surface of several types of immune system cells, including multinuclear leukocytes, macrophages, T cells, and splenocytes [9]. Opioids added directly to certain types of immune cells in vitro change the protein expression profile [14] and their function: they reduce macrophage chemotaxis and phagocytosis and weaken B-cell proliferation and antibody production [15]. In contrary to exogenous opioids, data on endogenous opioids are not clear, and however there are some studies suggesting the immunostimulating effect. Mathews et al. showed an increase in NK cells activity in response to beta-endorphin, which was reversible by naloxone [16].

A number of potential mechanisms of opioid-induced immunosuppression have been identified in experimental studies. It turns out that these drugs may reduce the number of macrophages available to fight infections [17] and weaken leucocyte migration as well as and peritoneal macrophage phagocytosis [18]. They may also interfere with respiratory burst activity, chemotaxis and superoxide production from neutrophils and macrophages [19], and immune cell recruitment to the wound site, which, in turn, may lead to impaired wound healing [20]. Studies show that opioids may also impair leucocyte endothelial adhesion by weakening intracellular adhesion molecules [21] and intensifying the apoptosis of macrophages [22] and T cells [23], which results in the impairment of the host defence barrier.

A number of mechanisms via which opioids impair adaptive immunity have also been studied. It turns out that these drugs may impair the T-cell viability and their proliferative response [9], T-helper cell function, and macrophage activity [9]. Moreover, opioids may also adversely affect humoral immunity by impairing primary antibody response (B cells) [24] and B-cell mitogenic response to bacterial liposaccharides (LPS) [25].

Another potential opioid-induced immunosuppression pathway is the hypothalamic-pituitary-adrenal (HPA) axis: the corticotrophin releasing hormone (CRH) stimulates the anterior part of the pituitary gland to produce adrenocorticotrophic hormone (ACTH), which, in turn, activates the adrenal cortex to produce glucocorticoids. The latter influences various components of the innate and adaptive immune system, suppresses cellular immunity, and contributes to the tolerance of various antigens by altering T- and B-cell function [26]. The effects of opioids on the HPA axis and its components (ACTH and glucocorticoids) are complex, species dependent, and time dependent and vary after acute and chronic administration [26]. Studies show that, in humans, acute administration of opioids results in a reduction or no change in ACTH or glucocorticoid levels [27]. On the contrary, chronic opioid administration may suppress the HPA, which may lead to adrenal insufficiency [10, 27]. Case reports have documented adrenal insufficiency after oral [28] or transdermal [29] opioid application. Additionally, chronic opioid injections are usually accompanied by disturbances in the daily rhythm of ACTH and cortisol secretion [27].

Studies have shown that opioids also affect the sympathetic system and by activating it, may cause immunosuppression. It is manifested, among others, by depressed NK cell activity and suppressed peripheral blood lymphocyte proliferation [30]. Felten et al. examined and described the contribution of the sympathetic system to the alteration of opioid-induced immune function and concluded that the phenomenon is caused by the rich sympathetic innervation of lymph nodes [31]. Other studies also validate this mechanism by indicating that the immune status is changed by opioids following sympathetic activation results from the rich adrenergic innervation of the spleen, lymph nodes, and bone marrow [30]. Moreover, both *α* and *β* adrenergic receptors have been identified in experimental studies on lymphocytes and macrophages in rodents [32].

Not all opioid drugs share the same immune profile. Some opioids seem to have no effects on immune function, whereas others tend to be immunosuppressive. This is probably due to the combination of direct effects on immunocytes and indirect effects in vivo that involve centrally mediated mechanisms as well as the systemic production and release of immunomodulatory mediators. Studies suggest that morphine has the highest immunosuppressive potential, fentanyl ranks medium, while buprenorphine and tramadol show the lowest or no immunosuppressive effect [33]. Morphine, codeine, and fentanyl suppress NK cell activity [3]. Hydromorphone and oxycodone, unlike morphine and fentanyl, do not impair the immunological functions, although they are more potent opioids than morphine [34]. Buprenorphine, which is a partial MOP opioid receptor agonist, has no immunosuppressive effect and does not impair the activity of NK cells and, hence from the immunological point of view, it is the safest opioid [35].

A list of opioids broken down by the degree of the immunosuppressive effect they induce is given in Table 1.

## 4. How Can Surgery and Perioperative Opioids Contribute to Cancer Progression?

Several possible mechanisms which are jointly responsible for cancer progression in the perioperative period have been identified. Numerous studies have confirmed that surgical manipulation, tumour dissection, and resection cause cancer cells to migrate to the cardiovascular system and their subsequent spread throughout the body [36]. Additionally, surgical intervention activates and intensifies the proliferation of neoplastic cells, inhibits their apoptosis [37], and stimulates their mobility, invasiveness, and adhesion—features considered very important in terms of the capacity of neoplastic cells to reach distant organs and metastasize [38]. It also promotes angiogenesis and networking of blood vessels, which offer cancer cells a kind of pathway to the host cardiovascular system and then further to the metastatic sites. The mechanism of neoplastic neoangiogenesis involves the vascular endothelial growth factor (VEGF), whereas other mediators, such as matrix metalloproteinases (MMPs), intensify adhesion and aid cancer cells in settling in distant organs [39].

The findings also suggest that the choice of the surgical technique also appears to be important. Less invasive options, including laparoscopic ones, demonstrate a less intense immunosuppressive effect compared with laparotomy [40]. Other factors of proven significance in increasing the risk of cancer progression are perioperative blood loss and blood product transfusions, especially kept in long-term storage [41], and hypothermia, which leads to intensified glucocorticosteroid secretion as well as impaired cellular and humoral immune response [42].

Stress and pain, unfortunately often experienced by patients in the perioperative period, also constitute important factors. They lead to stress hormone bursts and impair cellular immunity, including NK cell activity [43, 44]. The most important known factors responsible for cancer progression in the perioperative period are listed in Table 2.

Numerous studies suggest that opioids may promote cancer progression. The main mechanisms responsible for this adverse effect include the stimulation of angiogenesis and immunosuppression. Opioids, by activating MOP (*µ*) opioid receptors located on vascular endothelial cells, activate and promote angiogenesis and networking of new blood vessels, which play an important role in the transfer of cancer cells from the primary lesion site to the host cardiovascular system and further to the metastatic lesion sites. This mechanism involves, among other things, the vascular endothelial growth factor (VEGF) [33, 45]. Studies by Lennon et al. have confirmed that opioids stimulate the migration and proliferation of endothelial cells, including vascular endothelial cells via the vascular endothelial growth factor (VEGF) [46]. This mechanism involves the MOP (*µ*) receptor, since the administration of methylnaltrexone, which is a peripheral antagonist of the MOP receptor, reduces angiogenesis. Moreover, methylnaltrexone administered in combination with bevacizumab, which is an anti-VEGF monoclonal antibody, shows synergy in the inhibition of VEGF-induced angiogenesis [47]. Gupta et al. have shown that breast cancer cells implanted into mice grow and multiply much faster and show intensive neovascularization after the experimental animals have been exposed to morphine [48].

**Figure 2:**
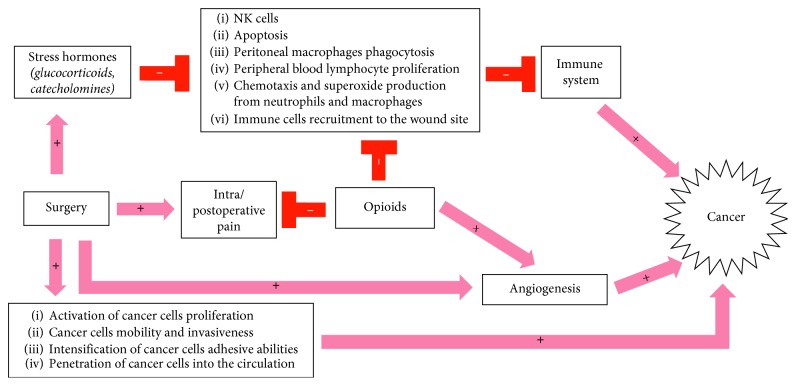
Perioperative immunosuppression mechanisms [16–23].

Opioids may lead to progressive immunosuppression via many of the mechanisms described in the previous part of the article. Studies show that fentanyl, an opioid frequently used intraoperatively, blocks the cytotoxic effect of NK cells in the postoperative period, whereas sufentanyl and alfentanyl, apart from their effect on NK cells, further weaken mitogen-induced lymphocyte multiplication [49].  Figure 2 shows the most important mechanisms of opioid-induced cancer progression.

## 5. Perioperative Setting: Possible Benefits of Regional Analgesia and Multimodal Analgesia

The increasing awareness of the immunosuppressive properties of opioids has prompted sustained efforts in search of other effective, but most importantly, safer methods of perioperative analgesic management. Regional analgesia techniques raise hopes in this respect. Their beneficial mechanism of action is complex: on the one hand, they provide effective analgesia, thus minimising the adverse effects of pain on the body of the operated patient and reducing the necessary doses of systemic analgesics (including opioids), which translates into improved safety of the therapy in terms of opioid-induced immunosuppression and potential risk of cancer progression. On the other hand, these techniques minimize the adverse effects of the surgical procedure itself by attenuating of the metabolic, neuroendocrine, and cytokine stress response to surgery and reducing the potential immunosuppressive and prognostic impact of stress and surgical trauma.

A number of studies have assessed the impact of regional analgesia techniques on cancer progression. The interpretation of their findings is difficult, since the surgical injury itself, ineffective pain treatment, and perioperative stress all exhibit immunosuppressive effects and increase cancer progression risk. Moreover, most studies assess the impact of regional analgesia techniques on patients who have additionally received general anaesthesia for surgery, yet, as is well known, numerous anaesthetics administered either intravenously or inhalationally also produce immunosuppressive effects. However, it does not alter the fact that the appropriately chosen and performed regional anaesthesia techniques (for a specific patient and surgical procedure) very effectively relieve pain and effectively block the surgical trauma-induced neuroendocrine response of the body and thus better preserves the patient's immune status. In a study done by Koltun et al., it has been seen that patients receiving epidural anaesthesia during colectomy had lower plasma levels of epinephrine and cortisol, compared to patients receiving general anaesthesia, and also the NK cell activity was better preserved in patients receiving regional anaesthesia [50].

Most of the studies in question assess the influence of central blocks (including continuous epidural anaesthesia) in prostate [51] and colorectal cancer surgery patients [52, 53]. The effects of the paraspinal block in breast cancer patients undergoing mastectomy were also assessed [54]. The findings of the studies are not unequivocal: some of them suggest a positive influence of regional anaesthesia techniques on immunological parameters (assessed differently in individual studies, e.g., VEGF, TGF-*β*, and IL-1*β*) and on the reduction of the risk of cancer progression, including prolonged overall survival time in patients in whom these techniques were used, whereas other studies fail to confirm these results [53, 55].

The currently recommended optimal approach to pain management is the multimodal analgesia approach, which involves combining analgesics and anaesthetic techniques with different mechanisms of action in order to take advantage of their synergistic effects. One of the components of multimodal analgesia are regional anaesthesia techniques: continuous epidural anaesthesia, paraspinal blocks, plexus blocks, and interfascial blocks which are increasingly often used in everyday clinical practice, thanks to the recent developments in ultrasonography. In the event of contraindications to regional anaesthesia techniques, combination pharmacotherapy involving nonopioid drugs and coanalgesics is a recommended analgesic management method (e.g., with intravenous lidocaine, magnesium, dexamethasone, ketamine, and dexmedetomidine). Such an approach, thanks to the combination of drugs with different mechanisms of action, ensures satisfactory analgesia at lower opioid doses, which is an important aspect of safety of the latter group of drugs referred to a number of times in this paper. Experimental studies show that NSAIDs administered together with opioids reduce immunosuppression associated with the use of the latter [54]. However, it should be remembered that the use of NSAIDs is subject to a number of restrictions due to the risk of gastrointestinal and renal damage, increased cardiovascular risk, and increased risk of bleeding secondary to the antiplatelet effect of nonselective NSAIDs. However, these drugs are worth considering as therapeutic options in cancer patients (taking into account the contraindications to their use), especially due to the NSAIDs' preventive effect on certain types of cancer and the possible anticancer effect of this group of drugs [56].

It should also be emphasized that the development of effective therapeutic strategies requires a better understanding of cellular mechanisms underlying the pathogenesis of pain. Progress in pain research points to an important role of microglial cells in the development of pain. The inhibition of spinal microglia has been shown to attenuate postoperative pain as well as morphine-induced antinociceptive tolerance [57]. Targeting microglial signaling might lead to more effective treatments for pain partly via improving the analgesic efficacy of opioids [58–60].

## 6. Conclusions

Insufficiently treated pain and stress associated with cancer adversely affect the body's defence mechanisms, including cellular immunity, natural killer (NK) cell function, and humoral response. Experimental and clinical studies also show the importance of effective pain management for checking cancer progression. Apart from the basic humanitarian aspect, these data provide an argument for the need to effectively alleviate stress and suffering in cancer patients. The question of how to do it optimally and in the safest possible way is still relevant, given the substantial evidence concerning the potentially adverse opioid effects to have emerged in recent studies (including immunosuppression, increased risk of infection, and cancer progression).

It should be clearly emphasized that the findings of research on the latter issue are ambiguous and as such should be treated with great caution. They must not be interpreted as an argument against using opioids in pain management in cancer patients. Effective pain treatment, alleviation of suffering associated with cancer, and improvement in the quality of life should always be a priority, while the awareness of potential risks should prompt the use of regional analgesia techniques, using local analgesics and possibly coanalgesics. Where it is impossible, recommendations include combined systemic pharmacotherapy comprising nonopioid analgesics and coanalgesics, some of which (including lidocaine and NSAIDs) also exhibit proven oncologically beneficial effects. Such an approach contributes to the optimization of analgesic management, and thus to obtaining the best analgesic effects at lower opioid doses, which, in turn, reduces the frequency and intensity of adverse effects associated with their use. Further research is needed to verify the currently available data on both the potential adverse effects of opioids and the possible protective effects of regional analgesia techniques on cancer patients.

## Figures and Tables

**Table 1:** Opioids and their immunosuppressive effects [33–35].

Strong immune modulation	Weak or no immune modulation
Morphine	Buprenorphine (the least or no effect)
Fentanyl	Oxycodone
Sufentanyl	Hydromorphone
Codeine	Tramadol

**Table 2:** Perioperative factors affecting cancer progression.

I. *Surgical factors*
** **1. Cancer cells enter the cardiovascular system during surgical procedures, including tumour resection, and subsequently spread across the body [36].
** **2. Surgical procedures activate and intensify the proliferation of cancer cells, stimulate their mobility and invasiveness, and increase their capacity for adhesion [37, 38].
** **3. Surgical factors inhibit the apoptosis of cancer cells [37].
** **4. Angiogenesis and the network of newly created blood vessels constitute a kind of “pathway” for the transfer of cancer cells to the host cardiovascular and further to metastatic lesion sites [39].
** **5. The selection of the surgical technique: less invasive techniques, including laparoscopic ones, have a less intense immunosuppressive effect compared with open techniques [40].
II. *The transfusion of blood and blood products*, especially those kept in long-term storage [41].
III. *Hypothermia* leads to glucocorticosteroid burst and impaired immune cellular and humoral response [42].
IV. *Stress and pain*, often experienced by patients during the perioperative period, cause stress hormone bursts and impair cellular immunity, including the activity of NK cells [43, 44].
VI. *Anaesthetic management*: type of anaesthesia used (general and regional), choice of drugs, especially opioids [50–55].
